# Molecular architecture of silk fibroin of Indian golden silkmoth, *Antheraea assama*

**DOI:** 10.1038/srep12706

**Published:** 2015-08-03

**Authors:** Adarsh Gupta K, Kazuei Mita, Kallare P. Arunkumar, Javaregowda Nagaraju

**Affiliations:** 1 Centre of Excellence for Genetics and Genomics of Silkmoths, Laboratory of Molecular Genetics, Centre for DNA Fingerprinting and Diagnostics, Hyderabad 500001, India; 2 State Key Laboratory of Silkworm Genome Biology, Southwest University, Chongqing 400716, China; 3 Deceased.

## Abstract

The golden silk spun by Indian golden silkmoth *Antheraea assama,* is regarded for its shimmering golden luster, tenacity and value as biomaterial. This report describes the gene coding for golden silk H-fibroin (AaFhc), its expression, full-length sequence and structurally important motifs discerning the underlying genetic and biochemical factors responsible for its much sought-after properties. The coding region, with biased isocodons, encodes highly repetitious crystalline core, flanked by a pair of 5′ and 3′ non-repetitious ends. *AaFhc* mRNA expression is strictly territorial, confined to the posterior silk gland, encoding a protein of size 230 kDa, which makes homodimers making the elementary structural units of the fibrous core of the golden silk. Characteristic polyalanine repeats that make tight β-sheet crystals alternate with non-polyalanine repeats that make less orderly antiparallel β-sheets, β-turns and partial α-helices. Phylogenetic analysis of the conserved N-terminal amorphous motif and the comparative analysis of the crystalline region with other saturniid H-fibroins reveal that AaFhc has longer, numerous and relatively uniform repeat motifs with lower serine content that assume tighter β-crystals and denser packing, which are speculated to be responsible for its acclaimed properties of higher tensile strength and higher refractive index responsible for golden luster.

Silk is a remarkable proteinaceous biomaterial, which is a unique possession of arthropods. Though silks are produced for an enormous number of purposes, holometabolous insects secrete a silken cocoon to encase their metamorphosing pupae, as a significantly strong selection factor. The cocoon silk of the domesticated silkworm, *Bombyx mori,* is globally renowned and its principal protein, fibroin is extensively studied. X-ray diffraction studies showed the presence of β-sheets in fibroin that are formed by the stacking of reiterated short arrays composed of small amino acids^1^,^2^. Lepidopteran larvae secrete silk from a pair of tubular secretory glands called silk glands, which are demarcated into posterior (PSG), middle (MSG) and anterior (ASG) regions that exit through a spinning orifice^3^. *B. mori* silk fiber has a fibrous core made of three elementary polypeptides, a fibroin heavy chain (H-fibroin or Fhc) of ~390 kDa, a fibroin light chain (L-fibroin or Flc) of 30 kDa which makes heterodimers and six such dimers interact with a glycoprotein, P_25_ to form 2.3 MDa elementary structural units of the fibrous core of silk, which in turn is multiply tunicated with glue proteins called sericins^4^,^5^,^6^,^7^. However, the fibrous core of silk secreted by wild silkmoths (family *Saturniidae*) is solely composed of H-fibroin with stretches of polyalanine that makes homodimers^2^,^8^. Based on X-ray diffraction studies, silk fibers characterized by the presence of strings of alanine were classified under group *3a* with an intersheet packing of 10.6 Å^9^.

Indian golden silkmoth, *Antheraea assama* (family Saturniidae) is semi-domesticated with a narrow habitat range confined to Brahmaputra valley of northeast India. *A. assama* is commonly called muga silkworm which spins golden cocoon silk, culturally acclaimed as a special product of India and the most expensive of silks^10^. It is highly valued in textile industry and in designing novel biomaterials for its unique biophysical properties like golden luster, tenacity and high absorbance of UV radiation^11^,^12^.However, extensive rearing and prospects of global recognition are deterred by the moth’s semi-domestic nature and extremely confined geographical distribution.

As the major component of silk fiber, the structure of H-fibroin determines its physical properties, which in turn are dictated by the type of the composite amino acids and their pattern of arrangement in full length. Determining full length gene sequence is significant to understand the role of each protein structural unit in the big picture. The sequences responsible for specific properties of interest allow engineering of better chimeric genes to refine the biophysical properties of fiber to spin composite silk fibers with better mechanical properties and to overcome the problems of endogenously expressed wild silks^13^. Sequence data of complex genes like H-fibroin allows the understanding of its relative status among similar genes and its adaptive trajectory in evolution. They also are important models of study for unusual evolutionary events like genetic polymorphism and accumulation of repetitive units by duplication through unequal crossing-over^14^. The similarity in evolution of repetitive region with that of the microsatellites evolution could be responsible for their clonal instability, making it formidable to characterize the complete structure of a full length H-fibroin^8^.

In order to explain the genetic and biochemical factors responsible for its properties, this report describes the comprehensive structure and expression of *A. assama* fibroin (*AaFhc*) gene that encodes H-fibroin protein. Silk gland specific transcriptomic sequence data from WildSilkBase EST library and in-house generated cDNA library were used to identify partial sequences of H-fibroin^15^. Full length H-fibroin gene was amplified through genomic PCR based on terminal sequence conservation and was cloned for determination of the complete sequence by constructing a sub-clone library of unique loci derived from separate digestions with different restriction endonucleases. The full length sequence was analysed *in silico,* to determine bias in usage of isocodons of its major amino acid residues, their composition in conceptually translated coding sequence, motif-assembly and fine repetitious organization of these motifs to predict secondary structure responsible for its remarkable properties and to study the evolutionary divergence of AaFhc from other H-fibroins. In addition, the report also describes the structure of *A. assama* silk gland and its cell enumeration details.

## Results and Discussion

### Silk gland structure

**Figure 1:**
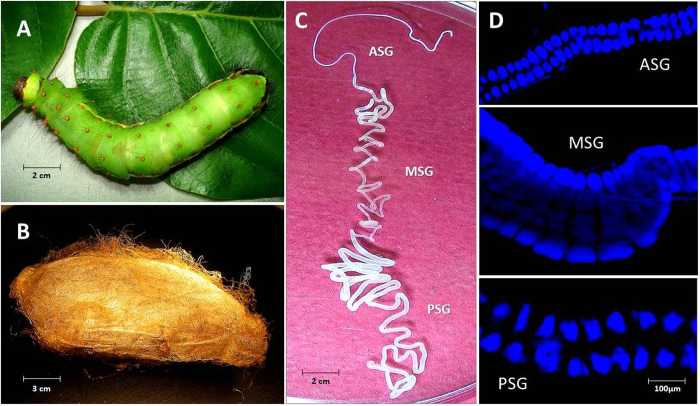
*Antheraea assama* (Saturniidae) larva, cocoon and silk gland. (**A**) Pre-spinning V instar feeding larva (about 8 cm long). (**B**) Golden silk cocoon (chrysalis, about 5 cm long). (**C**) One in a pair of silk glands (about 30 cm long) dissected from V instar pre-spinning larva of *A. assama*, placed in a Petri plate with 1X PBS. It shows features of a typical lepidopteron silk gland with three distinct regions: ASG (Anterior Silk Gland), MSG (Middle Silk Gland), and PSG (Posterior Silk Gland). (**D**) Fluorescent micrographs of portions of ASG, MSG and PSG of II instar larval silk gland showing stacks of paired glandular epithelial cells (DAPI stained) that makeup the cross-sectional circumference of silk gland.

Silk is synthesized in a pair of modified labial glands called silk glands. Each gland is composed of single-cell layered glandular epithelium in a long tubular structure enclosing a lumen made by stacking of just two secretory cells^3^. In *B. mori*, PSG is composed of about 525 secretory cells whose number remains constant, once determined in stage-25 of its embryogenesis^16^,^17^. The cell number is highly significant as it is a factor of its secretory activity. Silk glands of *A. assama* (Fig. 1A) secrete golden silk cocoon (Fig. 1B) at the end of larval stage. The ASG is about 5 cm long containing ~320 cells; MSG is about 10 cm long with ~550 cells, while the PSG is about 15 cm having ~800 cells surrounding luminal liquid silk (Fig. 1C,D). The PSG cells of *A. assama,* which are 35% higher than *B. mori* PSG, may cumulatively account for the larger cocoons in *A. assama* whose cocoon shell’s mean weigh is about 600 mg, almost twice the mean weight of a typical *B. mori* cocoon shell.

### Isolation and cloning of full length gene

**Figure 2:**
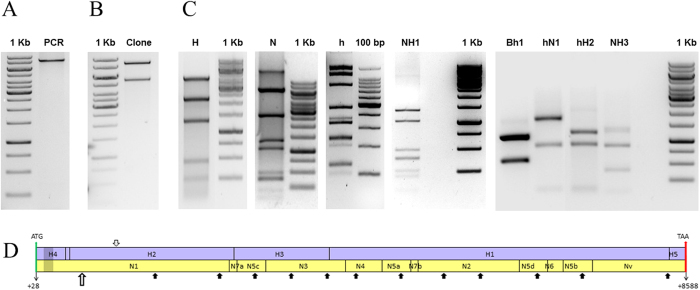
Full length PCR product, clone and restriction pattern of *AaFhc* gene. (**A**) Full length genomic PCR product of *AaFhc* (*lane* PCR; ~8.5 Kb) loaded against 1 kb DNA ladder; *lane* 1 kb (Thermo Scientific). (**B**) EcoRV digestion-released *AaFhc* gene; upper band corresponds to the insert fallout and the lower one corresponds to its backbone vector, pWKS30. (**C**) Restriction digestion pattern; *lane* H corresponds to the digestion of *AaFhc* with HhaI, *lane* N is NotI digestion of *AaFhc*–pWKS30 plasmid, *lane* h is the digestion of *AaFhc* with HaeIII, electrophorosed on 4% agarose gel against 100 bp DNA ladder, *lane* 100 bp (TaKaRa Bio Inc.), *lane* NH1 corresponds to H1 fragment digested with NotI, *lane* Bh1 is BamHI digested h1, *lane* hN1 is HaeIII digested N1, *lane* hH2 is HaeIII digested H2, and *lane* NH3 is NotI digested H3. (**D**) Restriction map of *AaFhc* gene; mauve strip represents HhaI restriction map and yellow strip is NotI restriction map. Transcription start is indicated with +1, while green and red arrows pointing to top represent the positions of translation start and stop codons. Block arrows mark HaeIII sites outside NotI restriction sites, while the grey and hollow arrowheads mark Bsu36I and BamHI restriction sites respectively. Dull patch towards 5′ end within H4 and N1 marks the position of intron. Gels were run under same experimental conditions and displayed in cropped format.

Standalone BLASTn on PSG specific transcriptomic sequences yielded sequences homologous to H-fibroin gene of *A. pernyi* among which most of them map to non-repetitious ends. The rest of the matched contigs encode repeat region of H-fibroin with good alignment mainly due to conserved polyalanine repeat motifs. The contigs corresponding to the repeat region of H-fibroin are not useful for foolproof assembly to obtain complete sequence, and do not harbour unique primer binding sites for amplifying partial or intermittent sequences^18^. AaFhc is rich in alanine codons (GCX), besides being a highly expressed gene, invariably making it GC-rich. Such GC-rich regions are less susceptible to shearing, and remain as long strands, which have very less efficiency towards adapter mediated blunt-end cloning, eventually escape the transcriptome^19^. Therefore, an end-to-end long PCR was performed to amplify full length *AaFhc* gene. Long PCR was performed on genomic DNA extracted from a single pupa to avoid polymorphic variations. Electrophoresis of PCR product revealed a single sharp band of about 8.5 kb (Fig. 2A). Complex repetitious genes like fibroin exhibit sequence instability through rearrangement events and deletions caused by unequal crossing over, often termed as exon shuffling, which has evolutionary importance for introducing variations^14^. Such genes are often termed as Coding Microsatellite Sequences, which are also known for their clonal instability in heterologous systems^20^. Hence, the PCR-amplified full length gene was cloned (Fig. 2B) and maintained in SURE strain of *E. coli*. Despite the promising properties of silk, not many silk fibroins are fully characterized. The greatest challenge in full length sequencing is their repetitious nature and clonal instability.

### Subcloning strategy for full length sequencing of AaFhc gene

The conventional technique of primer-walking is not useful to determine the full stretch of intermittent repetitious region. Hence, subcloning strategy was followed for full length sequencing of the repetitive region of *AaFhc*. Restriction digestion based library of 64 sub-clones was generated by separate complete digestion with different restriction endonucleases of both *AaFhc* full length fallout and the resultant large fragments with a frequent cutter restriction enzyme. Restriction enzymes with GC rich recognition sites were identified and individually checked for their pattern and frequency of restriction on full length gene. Digestions of full length *AaFhc* with restriction endonucleases with GC-rich recognition sites: HhaI, NotI, and HaeIII resulted in sub-clones (Fig. 2C) named H, N and h respectively. HhaI digested *AaFhc* resulted in 5 prominent bands: H1 (4473 bp), H2 (2178 bp), H3 (1266 bp), H4 (408 bp), and H5 (215 bp). NotI digestion of *AaFhc*–pWKS30 plasmid resulted in pWKS30+Nv, which is linearised plasmid with a portion of *AaFhc* 3′ end (5433 bp + 1222 bp), N1 (2429 bp), N2 (1323 bp), N3 (1053 bp), N4 (480 bp), N5 band has four co-migrants (N5a-d: 381 bp, 378 bp, 375 bp and 372 bp), N6 (207 bp), and N7 has two co-migrants (N7a-b: 108 bp and 102 bp). HaeIII digested *AaFhc* resulted in h1 (1585 bp), h2 (1220 bp), h3 (846 bp) h4 (has 2 co-migrants: h4a of 480 bp and h4b of 474 bp), h5 (375 bp); h6 (has 3 co-migrants, h6a-c: 343 bp, 341 bp and 337 bp), h7 (has 5 co-migrants, h7a-e:250 bp, 247 bp, 244 bp, 241 bp, and 236 bp); h8 (207 bp); h9 with 7 co-migrants, h9a-g (139 bp, and 6 fragments of 131 bp); h10 has 2 co-migrants, h10a-b (108 bp and 102 bp). Further, the fragments of digestion and secondary digestion were cloned (in pWKS30) and sequenced by priming vector-borne M13 sites to obtain full sequence of clones of about 1 kb. The larger fragment clones were subjected to further digestion with NotI and HaeIII. NotI digested H1 fragment resulted in NH1-1 (1323 bp), NH1-2 (1007 bp), NH1-3 (480 bp), NH1-4 (=N5a), NH1-5 (223 bp), NH1-6 (207 bp), and NH1-7 (102 bp). BamHI digested h1 fragment resulted in Bh1-1 (1093 bp) and Bh1-2 (492 bp). HaeIII digested N1 resulted in hN1-1 (=h1), hN1-2 (=h3) and hN1-3 (129 bp). H2 was digested with HaeIII as it lacks NotI site, resulted in hH2-1 (1153 bp), hH2-2 (131 bp), and hH2-3 (48 bp). NotI digested H3 resulted in NH3-1 (830 bp), NH3-2 (378 bp), and NH3-3 (58 bp). The digested fragments derived from most recent digestion were named first and numbered in descending order of their size, (e.g., NH1-2 is the second largest fragment resulted upon NotI digested H1 sub-clone, whereas H1 is the largest fragment resulted upon HhaI digestion of *AaFhc*), while the co-migrants are designated with alphabets in lower case. The pattern of arrangement of NH1 sub-clones within H1 was determined by partial digestion of H1 with NotI. Overlap of sequences eventually generated a restriction map of the full length gene. Individual sub-clone sequences were confirmed for their orientation by checking their characteristic repeats upon conceptual translation. The restriction map of full length gene was in agreement with the electrophoretic restriction pattern of HhaI and NotI sub-clones falling on common loci and account for same cumulative size.

### AaFhc gene expression

**Figure 3:**
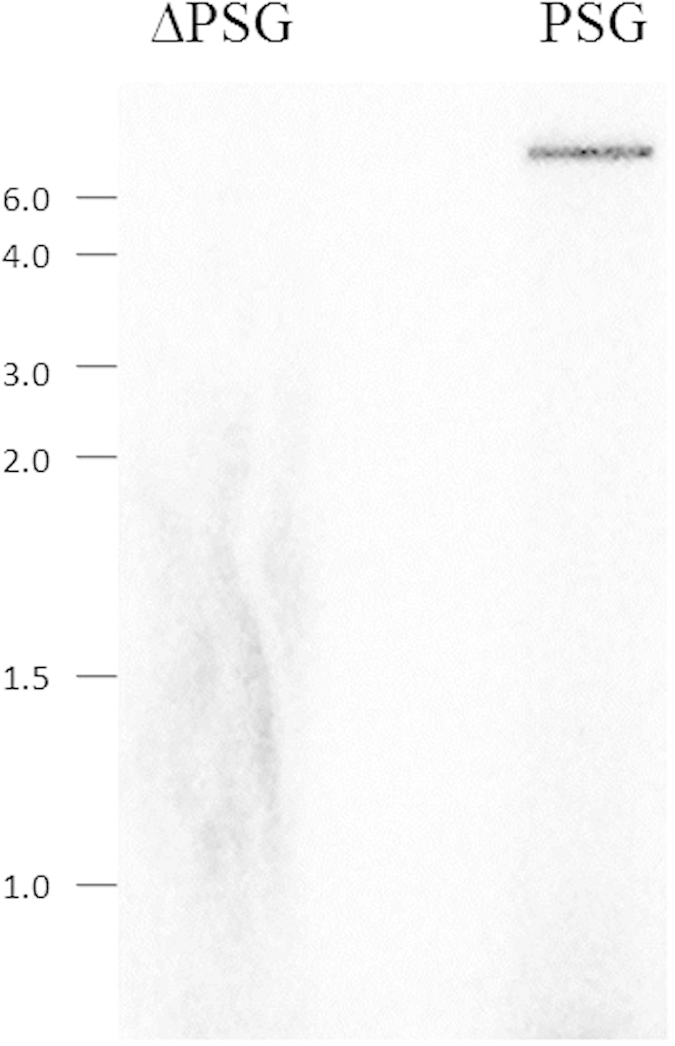
Expression of *AaFhc*. Northern blot analysis shows strict tissue-specific expression. *lane* PSG: poly(**A**) RNA prepared from posterior silk gland. *lane* ∆PSG: whole larval carcass only without posterior silk gland.

Northern blotting was performed to analyze the expression of *AaFhc*, which showed a single band only in PSG lane of size ~8.5 kb (Fig. 3). Specificity of territorial expression in PSG was confirmed by loading mRNA prepared from whole larval carcass except PSG region of silk gland (*lane* ∆PSG) as a negative control. The transcript size calculated from the northern blot corresponds to the gene size resulted in full length long PCR, and also suggests an estimated molecular weight of AaFhc protein to be around 250 kDa.

### Structure of AaFhc

The coding region, partial promoter region, intron and the UTRs of *AaFhc* have been deposited in the GenBank database under accession number KJ862544. Restriction mapping of *AaFhc* resulted in the full length genomic sequence of 8561 bp (ATG-TAA), which is intervened by a short intron towards its 5′ end. Its 8532 bases long transcript is stitched out of two exons and flanked by a pair of short untranslated regions (UTRs). Exon 1 is 69 bases long, with a short 5′ UTR of 27 bases and the coding region marked with the start codon, AUG. Exon 2 is 8385 bases long with the coding sequence of 8385 bases for 99.5% of the protein, followed by a short 3′ UTR. Non-essential amino acids makeup the major portion of AaFhc (92.8%), and highly reduced occurrence of essential amino acids (Arg, His, Leu, Ile, Met, Phe, Thr, Trp, and Val) is possibly due to the inability of insects to synthesize amino acids or obtain significant ration from the symbiotic flora, as synthesizing a proteinaceous cocoon exclusively through diet could have been highly expensive^21^.

**Figure 4:**
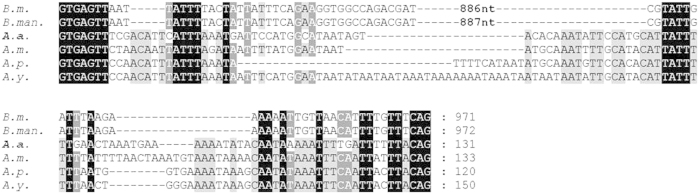
Conservation of H-fibroin intronic sequences of *B*. *mori* (*B.m.,* AF226688), *B. mandarina* (*B.man.,* X03973), *A. assama* (*A.a.,* KJ862544), *A. mylitta* (*A.m.,* AY136274), *A. pernyi* (*A.p.,* AF083334) and *A. yamamai* (*A.y.,* AB542805). *A. assama* H-fibroin sequence is placed in the middle for comparing Saturniids’ and Bombycids’ Fhc intronic sequences.

**Figure 5:**
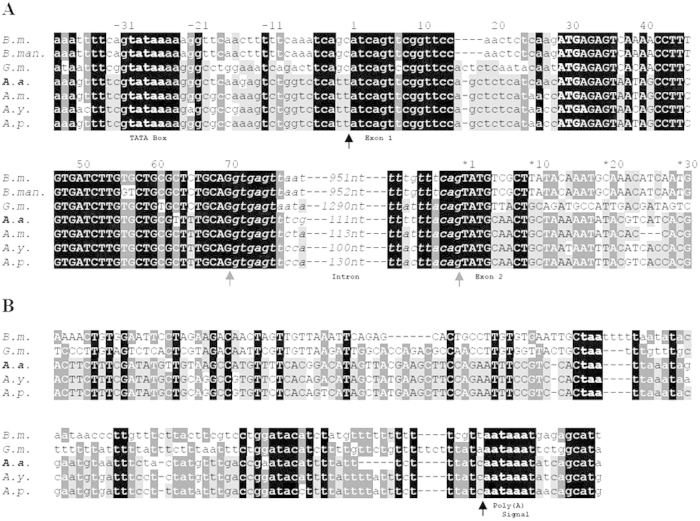
Alignment of 5′ and 3′ end sequences of H-Fibroin gene of *B.* *mori* (*B.m.*, AF226688), *B. mandarina* (*B.man.*, X03973.1), *G. mellonella* (*G.m.*, AF095239 and AF095240), *A. assama* (*A.a.*, KJ862544), *A. mylitta* (*A.m.*, AY136274), *A. yamamai* (*A.y.*, AB542805) and *A. pernyi* (*A.p.*, AF083334). *A. assama* H-fibroin sequence is deliberately placed in the middle for comparison with other saturniids and also other lepidopterans. Coding regions are represented in upper case, and the non-coding ones in lower case. Black arrowheads mark the transcription start and end, while the gray arrowheads mark the intron boundaries. Translation start codon is boldfaced. The nucleotides are numbered from each exon-start. (**A**) represents the aligned 5′ sequences and a stretch of upstream non-coding region of H-fibroin. Intron is represented in lower case and italicized. TATA motif and start codon are boldfaced. (**B**) the alignment shows coding and non-coding 3′ regions of H-fibroin. Stop codon and polyadenylation signal motif are boldfaced.

- *Intronic sequence –* Highly expressed genes are selected preferentially to have shorter and fewer introns to cut the cost of transcription and splicing^22^. Similarly, *AaFhc* gene has a small intron of 131 bases towards 5′ end and it is AT rich (20.6% GC). Its sequence and length are well conserved across Saturniid H-fibroins (Fig. 4). The sequence towards the ends and splice-junctions is conserved with much longer BmFhc intron, probably due to the presence of regulatory protein binding AT-rich elements of fibroin gene modulators^23^. Computational analyses revealed the presence of long palindrome sequences in intron but they do not assume stem-loop conformation required for miRNA synthesis.
- *Regulatory and untranslated regions –* Inverse PCR was performed to obtain promoter sequence. In order to achieve this, we took advantage of BamHI restriction site present 491 bp downstream of the translation start codon of *AaFhc*. Primers for iPCR were designed upstream to BamHI site, such that the forward primer anneals to intron and the reverse one to exon-1. The 319 bp long sequence resulted from iPCR was determined to be upstream of the translation start codon, which corresponds to part of its promoter region and 5′ UTR, whose sequence and +1 position of the exon 1 are conserved across lepidopteron *H-fibroins* (Fig. 5A). The partial promoter sequence has conserved protein binding sites for transcriptional regulation^24^. CAAT box and TATA box motifs are present at −224 and −25 positions respectively, upstream of +1 base of exon 1. Highly conserved regulatory motif for silk gland-specific transcription, TGTTT called Silk gland factor (SGF) is present upstream of −132 position^25^. The location of SGF motif in *A. assama* is similar to other H-fibroin promoter sequences, residing between CAAT box and TATA box motifs. 3′ RACE resulted in the sequence downstream of the translation stop codon. It includes a 78-nucleotide long 3′ UTR (Fig. 5B) starting with the translation stop codon, UAA (following +8454) and a polyadenine tail. Polyadenylation signal, AAUAAA is present 56 bases downstream of UAA.
- *Deduced amino acid sequence –* The first 18 residues in 127 amino acids of N-terminal amorphous motif are predicted to make secretory signal peptide, which is highly conserved across diverse silk spinners (Supplementary information 1). Downstream of the secretory cleavage site, six Asp residues are present in the N-terminal amorphous motif, the highest among all reported saturniid H-fibroins besides *A. yamamai* H-fibroin. Asn residues are not present anywhere else in the protein, which most-probably mediate N-terminal glycosylation. This motif is the least hydrophobic region (49.5% polar) of AaFhc (GRAVY value, −0.424). The unique feature of AaFhc is the presence of a hexaserine linker juxtaposing the N-amorphous motif and the crystalline core, unlike any other H-fibroin.

**Figure 6:**
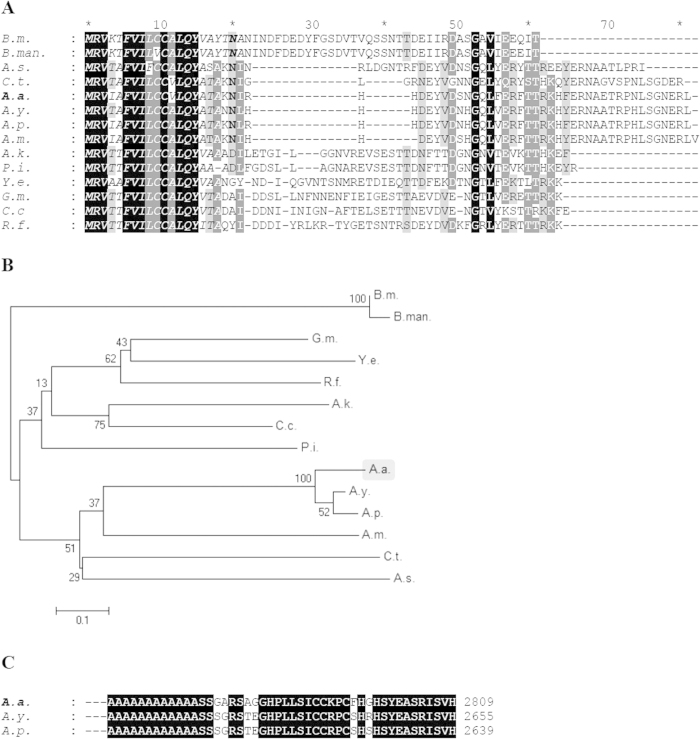
Conservation of terminal non-repetitive sequences of H-fibroin proteins. (**A**) the N-terminal sequences from H-fibroin of *B. mori* (*B.m.,* AF226688), *B. mandarina* (*B.man.,* X03973), *Actias selene* (*A.s.,* GU212855), *Cricula trifenestrata* (*C.t.,* JF264729), *A. assama* (*A.a.,* KJ862544), *A. yamamai (A.y.,* AB542805), *A. pernyi* (*A.p.,* AF083334), *A. mylitta* (*A.m.,* AY136274), *Anagasta kuehniella* (*A.k.,* AY253534), *Plodia interpunctella* (*P.i.,* AY253533), *Yponomeuta evonymellus* (*Y.e.,* AB195979), *G. mellonella (G.m.,* AH009792), *Corcyra cephalonica* (*C.c.,* GQ901977), *Rhodinia fugax* (*R.f.,* AB437258) are aligned to show conservation. Highest level of similarity is represented with black background and lesser ones with gray. Signal peptide sequences at N-terminal side are italicized. (**B**) Unrooted bootstrap consensus phylogenetic tree was generated from the amino acid multiple sequence alignment ‘A’, with bootstrap support values indicated at branch points. The bar represents the number of amino acid replacements per site and AaFhc highlighted with a gray background. (**C**) conservation at the non-repetitive C-terminal sequences of *Antheraea* H-fibroins.

N-terminal amorphous motif is the most conserved among all regions across all reported insect H-fibroins (Fig. 6A). Hence they were used for multiple sequence alignment as the rest of the sequence cannot be reliably aligned across the species for generating a cladogram for phylogenetic analysis. Phylogenetic tree resulted in clustering of all *Antheraea* specific H-fibroins into a distinct clade from other H-fibroins, wherein AaFhc stands out as a distinct branch among other *Antheraea* specific H-fibroins, with strong bootstrap support value on its clade suggesting its divergence (Fig. 6B).

The rearmost 34 residues of AaFhc encoded by the 3′ non-repetitive region constitute the C-terminal amorphous motif, which is highly conserved (82% similarity) among reported saturniid H-fibroins (Fig. 6C). It is dominated by alkaline residues (20.6%) that elevate its p*K* value to 8.66, which is highest within AaFhc. The unique feature of this motif is the presence of three Cys residues that are absent elsewhere, excluding the targeting peptide. They most probably mediate the formation of disulphide linkages in dimerisation. Serine content is higher in the C-terminal amorphous motif (20.6%) than in N-terminal amorphous motif (14.2%) and the crystalline core (9.9%), besides other polar amino acids (26.4% excluding Ser), which probably is important in attributing more solubility to the region for compensating the absence of a P25-like glycoprotein homolog^26^. Relative position of Cys residues and the sequence conservation of C-terminus most probably have evolutionary significance among saturniids, with respect to the formation of homodimers in making the elementary structural units of fibrous core of silk fiber.

### Repeat motifs of AaFhc

Non-repetitive termini flank the crystalline-core encoding repetitive region of 7794 bases that account for 92.5% of the coding region. The repetitive region makes a prominent CpG island in the full length gene with highest GC (about 65%) content within the coding sequence. It encodes the most hydrophobic region (GRAVY value, 0.186) of the entire protein (GRAVY value, 0.22) as 76.8% of it is composed of nonpolar amino acids, dominated by Ala (44.5%) and Gly (30.1%). The third most abundant amino acid in the crystalline core is Ser (9.9%), being lowest among all reported H-fibroins.

The crystalline core is an assemblage of arrayed short repeat motifs of 5 to 23 amino acids. AaFhc has the highest number of repeat motifs among all reported saturniid H-fibroins. The crystalline core of AaFhc has polyalanine stretches (A_5–15_) called A-motifs that alternate with other types of motifs called non-polyalanine motifs. A-motifs correspond to 37.4% out of the total Ala content (42.5%) of AaFhc, holistically making it Ala-rich in contrast to the Gly-rich BmFhc. A-motifs of AaFhc are absolutely devoid of other types of amino acid residues, unlike other reported saturniid H-fibroins, which are occasionally interrupted by serine residues that account for 3–4% of their polyalanine region.

**Figure 7:**
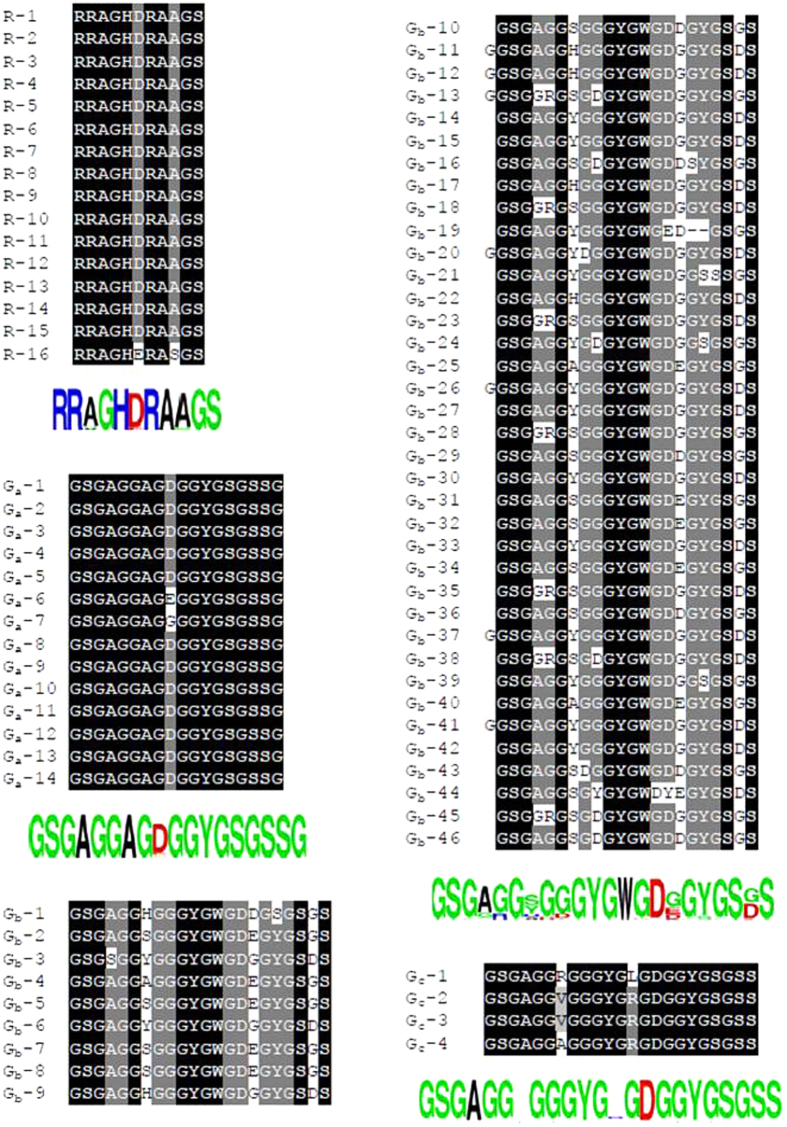
Conservation within non-polyalanine motifs of AaFhc. The repeat motifs are numbered according to their position in AaFhc molecule.

Based on the predominant amino acid residue, we classified non-polyalanine motifs into two categories: Arg-rich R-motifs and Gly-rich G-motifs (Fig. 7). R-motifs are the regions of remarkable conservation within the crystalline core of AaFhc, and across other saturniid H-fibroins, indicating differential evolutionary pressure along the sequence. The primary sequence of AaFhc has 17 R-motifs, which is the highest number among all reported saturniid H-fibroins. They are least hydrophobic in the crystalline core, as they are made up of about 54% polar amino acid residues (27% R and 9% S), higher than any other reported saturniid H-fibroins.

G-motifs make 53.4% of the crystalline region, which is highest among reported saturniids with higher concentrations of Gly (54.1%) and Ser (17.5%). However, the concentrations of Ala (5.5%), Tyr (9.2%) and acidic-basic amino acid ratio (0.16) are the lowest among G-motifs of other saturniids (Supplementary information 2). They exhibit the highest degree of polymorphism over other motif types of AaFhc. Based on the sequence variations and reorganization, G-motifs include three subtypes, G_a_, G_b_, and G_c_ motifs, which are 18 to 23 residues long. All G-motifs have homology at their proximal ends (GSGAGG), but differ in their intermittent residues, length and number. The rearrangement events like duplication and variation in G-motifs due to genetic instability conferred by Chi-like sequence is viewed as an adaptive variation in response to natural selection^27^. Howsoever instable, they are highly conserved within the sequence and across species suggesting a functional constraint in their evolution^28^. But when compared with other saturniids, the G-motifs of AaFhc show highest degree of sequence conservation among themselves and in the uniformity of their length (Fig. 7). G_a_ is the shortest G-motif in AaFhc which is 18 amino acids long and 15 such are distributed in AaFhc. G_b_ motifs make up 38.4% of the crystalline domain, the most abundant non-polyalanine motif that repeats 46 times in AaFhc. No other H-fibroin is as much dominated with a single type as G_b_ does in AaFhc. G_c_ motif is the longest of all G-motifs, but is least populated. The G_c_ motifs present towards N and C-termini have a unique RGD tripeptide that is previously shown to have high value in designing potential biomaterials^29^.

**Figure 8:**
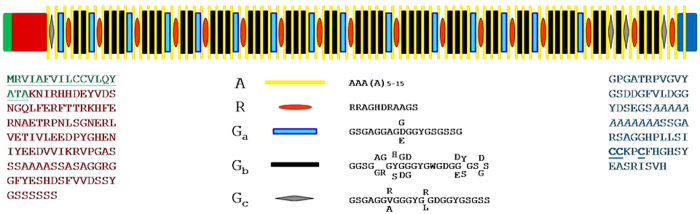
A model of AaFhc illustrating the arrangement of repeat motifs. The repetitious pattern of repeat motifs arrangement in AaFhc protein sequence is shown in the above schematic. Each type of motif is represented in different geometric shapes. The non-repetitious amino and carboxyl regions flanking the intermittent repeat region are shown as proportionately larger blocks. Signal peptide in the N-terminus is shown in green and its sequence is underlined, while the Cysteine residues in the non-repetitive C-terminus are bold-faced.

Increased uniformity in motif length suggests homogenization, similar to the repetitive regions of Flagelliform silk of golden orb-web spider, *Nephila clavipes*, known for its toughness and elasticity through increased orderliness resulting in tighter packing of crystal forming units^30^. This unique feature in AaFhc suggests comparatively fewer unequal cross-over and slippage events, probably due to higher selective stringency. Putative GGY tripeptide repeats 103 times in G-motifs, but is absent in a few G-motifs unlike any reported saturniid H-fibroin. However, a ubiquitous and the most prominent tripeptide, GYG is repeated 124 times in all 66 G-motifs, probably due to multiple events of replication slippage and unequal crossing-over during evolution^31^. The repeat motifs generally do not show a strict preference of pairing among themselves along the primary sequence, except that R-motif prefers to be coupled with G_a_-motif. Since R-motifs lack Chi-like sequence, they prefer docking with G_a_-motifs, except for R-16 and R-17, which are paired to G_c_3 and G_c_4 motifs, the only pair of G_c_ motifs that contain Chi-like sequence, unlike *A. pernyi* H-fibroin, where in all G-motifs harbor Chi-like sequences^8^. This peculiar type of adaptive flexibility in pairing with motif types signifies its importance in recombination. Thus, G-motifs play the most significant role in the structure, function and evolution of saturniid H-fibroins. The amino acid residues with long hydrophobic side chains such as Ile, Leu and Val are confined towards termini, and they constitute lowest proportion (1.2%) among all reported H-fibroins. The repetitious arrangement of motifs within the primary sequence of AaFhc is illustrated in the model (Fig. 8).

### Preferential codon usage

The coding sequence of *AaFhc* is GC rich (63.4%), specifically G-rich (40.3%), resulted due to abundance of codons of Ala (GCX) and Gly (GGX). It is observed that the most abundant residue of AaFhc, Ala (42.5%) is preferentially coded by GCA (67.3%), similar to the Ala-rich major ampullate (MA) silk in dragline of spiders. Whereas, the most preferred Ala-isocodon in the non-polyalanine motifs of AaFhc, GCU is similar to the Gly-rich BmFhc. The longest stretch of contiguous GCA trinucleotide in *AaFhc* is nine, which is higher than all reported saturniid H-fibroins. However, a few synonymous point mutations interrupt GCA concatenation in order to stabilize the tract and the size of A-motifs^8^. The most abundant amino acid in *B. mori* H-fibroin (BmFhc), Gly is the second most abundant (28.9%) in AaFhc, which however is highest among reported saturniid H-fibroins. Among its isocodons, GGU is the most preferred (41.6%, highest among all reported saturniid H-fibroins) and GGG is the least preferred (0.6%, lowest among all reported H-fibroins). AaFhc has the lowest amount of Ser (10.2%) among all reported H-fibroins (Table 1), but its most preferred isocodon, UCA has highest frequency (69.7%) among all reported H-fibroins. As a consequence, 2172 out of 2809 codons in *AaFhc* CDS (77.3%) have G in its first place (first C, 0.03%), and 1457 codons (51.86%) has C in its second place (second G, 34.3%). Therefore, the most preferred isocodons of the major composite amino acids, GCA (Ala), GGU (Gly) and UCA (Ser) make up 47.73% of *AaFhc* gene, which is the highest amount of codon bias among all reported saturniid H-fibroins. Strong bias for G and C in first two positions resulted in an equally strong bias for A/U in the third place of codons (77.1%) as counter-balance effect to check the overall GC content^32^. Dominance of particular amino acid type cumulatively constrained the usage of particular isocodon in its coding sequence resulting in codon bias (Table 2). Moreover, such biased preference for specific isocodons is significant in maintaining the stability of the mRNA secondary structure and in tRNA adaptation by large-scale transcription of few particular iso-tRNAs^30^,^33^.

### Secondary structure

**Figure 9:**
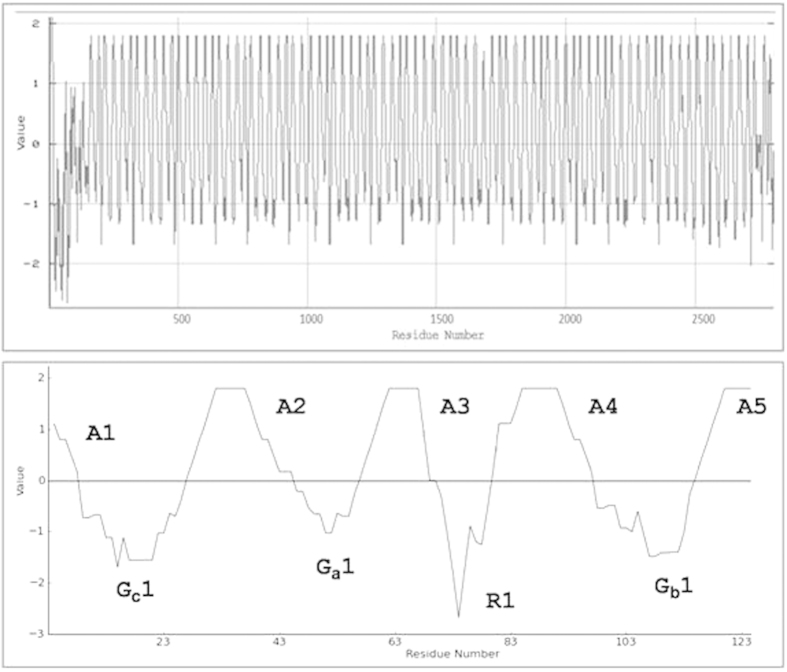
Hydropathy of AaFhc. Hydrophobicity/hydrophilicity of conceptually translated *AaFhc* coding sequence analysed. The upper panel represents the full length primary sequence while the lower box represents a portion (127 to 255 amino acid residues) to represent all repeat motif types in one stretch.

The sequence similarity of AaFhc with *A. pernyi* H-fibroin and polyalanine stretches of *Nephila* MA silk suggests a strong positive pressure in maintaining polyalanine motifs and the basic blueprint of its crystal-contriving domains, making it radically different from BmFhc. Regular interruption of the A-motifs with non-polyalanine motifs results in the sequential alternation of hydrophobicity in AaFhc (Fig. 9), due to lower hydrophobicity of non-polyalanine motifs that alternate highly hydrophobic A-motifs as shown in the hydropathy plot^34^. With the knowledge of full length sequence of AaFhc, predicting its secondary structure is crucial to elucidate its properties. Self-optimised prediction method (SOPMA) suggests that the protein makes 45% α-helices, 34% random coils, 14% β-turns and 6% extended β-strands^35^. A-motifs are predicted to be involved in making α-helices and G-motifs partially involved predominantly in making 3_10_ helices and β-turns that act like springs conferring elasticity, while R-motifs prefer making coils and β-turns within the crystalline core. The predicted conformation of polyalanines of AaFhc is in agreement with the NMR spectroscopic studies previously conducted on regenerated aqueous solution of *A. pernyi* H-fibroin, which also suggested preference of polyalanine sequences in making α-helices in aqueous solutions^36^. Whereas, polarized Raman micro-spectroscopic studies on fiber showed that the polyalanine stretches make antiparallel β-sheets through inter-molecular non-covalent interactions^37^. These intermolecular interactions that link β-sheets play a significant role in conferring tensile strength^38^.

A-motifs confer stronger hydrophobicity required for faster gelation and higher binding energy in the formation of stronger β-sheets between the crystalline repeats than (GA)_n_ β-sheets of BmFhc^36^,^39^. The crystallinity of A-motifs in AaFhc is speculated to be much higher than that conferred by short poly(Ala) repeats in MA silk of *N. clavipes*. Polyalanines as short as (A)_6_ are sufficient to make a strong β-nanocrystal^40^. This remarkable feature is responsible for the outstanding tensile strength of *A. assama* silk comparable to that of *B. mori* silk, and is highest among all reported wild silk fibers because of stronger polyalanine crystals uninterrupted by serine residues unlike other saturniid H-fibroins^41^. Previously conducted electron diffraction studies on *N. clavipes* MA silk showed that Gly-rich motifs preferentially make large β-sheet crystals but are less orderly than polyalanine β-crystals^42^. This feature in AaFhc can be attributed to the frequency of large aromatic residues (W: 3.3% and Y: 9.3%) in G-motifs, which affect the regularity in packing of crystalline units unlike GGX motifs of BmFhc. However G_a_ motifs have GGA tripeptide and few G_b_ motifs have GGS in them, which are involved in making α-helices. Thus, the occurrence of GGX (X: Ala/Ser) in G-motifs partially involved in α-helical structures could reorient the chain^39^. Tight β-crystals formed by A-motifs are responsible for the fiber tensile strength, while glycine rich sequences confer to the fiber elasticity^43^.

Elongation at break of *A. assama* silk (40%) is highest among all reported saturniid silks and is comparable to *N. clavipes* MA silk (40%), which is much higher than *B. mori* silk (15%), maybe because of unfolding of amorphous random coil regions^44^,^45^. Recent studies with FTIR spectroscopy revealed that H-fibroin of *A. pernyi* has highly oriented β-sheets and slightly oriented α-helices and random coils that are slightly oriented^46^. In light of the unique features of AaFhc, like less heterogeneity and orderly arrangement of non-polyalanine repeats in higher number, with lesser amount of polar residues, tighter polyalanine β-crystals that are devoid of non-alanine residues, lead to its higher hydrophobicity and highest ultimate tensile strength. Higher hydrophobicity seems to have an evolutionary advantage considering the insect’s habitat confinement to one of the wettest regions on Earth. Besides higher crystallinity, the fiber is denser due to compact packing and is less porous, resulting in highest value of isotropic refractive index (1.557) among all reported saturniids^44^. Conclusively, all these unique and interdependent intramolecular and intermolecular structural factors are speculated to be cumulatively responsible for the tensile strength and unique golden luster of *A. assama* silk fiber.

### H-fibroin protein of A. assama

**Figure 10:**
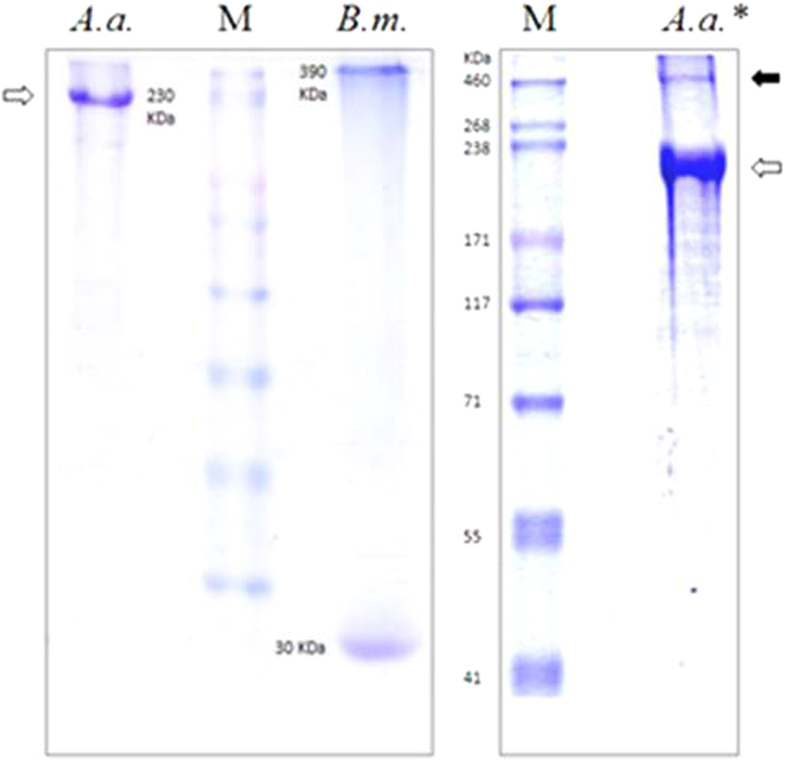
SDS-PAGE of degummed cocoon silk showing AaFhc. *A. assama* degummed cocoon silk showed single band of H-fibroin at 230 kDa, in lane *A.a.* marked with a hollow arrow head. In lane A.a.*, an extra band is visible at 460 kDa (solid arrowhead), besides the expected 230 kDa band, which corresponds to its uncleaved remnants of H-fibroin homodimer. Lane *B.m.* corresponds to degummed silk protein sample prepared from *B. mori* silk, used as positive control to show the absence of corresponding fibroin light chain homolog in *A. assama* degummed silk. Lane M corresponds to protein ladder showing heavy molecular weight size standards (460 kDa to 41 kDa).

Freshly spun cocoon was used for protein preparation to minimize degraded protein. Dried degummed cocoon weighs only 26.3% less than its original weight. Therefore, *A. assama* cocoon-silk is composed of 73.7% fibroin by weight. Fibroin solubilised in saturated LiSCN solution was solvent-exchanged to 8M Urea in order to minimize the anomalous in-gel mobility of proteins due to chaotropicity rendered by Li^+^ ions. Fibroin solution was resolved on SDS-PAGE, resulting in a single band as shown in the lane *A.a.* (Fig. 10). The molecular weight was calculated to be about 230 kDa, matching with the mass deduced from the conceptually translated CDS, excluding its signal peptide, 227.5 kDa. In lane A.a.*, an extra band was observed at 460 kDa, which corresponds to the uncleaved AaFhc homodimer in the cocoon silk, appeared probably due to incomplete reduction of disulphide linkages by β-Mercaptoethanol during sample preparation. Unlike BmFlc band seen in the positive control, lane *B.m.*, a band corresponding to it was absent in lane *A.a.*, which suggests the absence of fibroin light chain in *A. assama* silk. Silk gland specific cDNA libraries were BLAST searched in vain for *BmFlc* homologues, which also suggests the lack of its expression even in transcript level. All these evidences confirm that AaFhc makes a homodimer similar to other reported saturniid H-fibroins.

## Methods

### Insect rearing and sample preparation

Culture of fifth instar larvae was maintained at 25 °C on natural diet of fresh leaves of soalu, *Litsaea polyantha*. On fourth day after fourth molting, the larvae were dissected and silkglands were collected. The silkglands were gently rinsed in 1X PBS (Phosphate-Buffered Saline, 0.1 M Na_2_HPO_4_, pH=7.0, 0.15 M NaCl) prepared with DEPC-treated water (Ambion) and were carefully dissected for their posterior silk gland regions, discarding the tissue junctions. The tissues were snap-frozen in liquid nitrogen and stored at −80 °C before proceeding to RNA isolation. Fresh cocoons were cut open to collect pupae for genomic DNA isolation and the silken cocoon shell for fibroin protein preparation; both stored at −80 °C till further analysis.

### Silk gland cell enumeration

On the first day after the first molt, the larvae were immobilized on ice and dissected under stereomicroscope to collect silk glands. The silk glands were gently rinsed in 1X PBS and incubated with ice-cold 0.1% Triton X (in 1X PBS) for 10 min., rinsed with 1X PBS and fixed with 4% p-Formaldehyde (in 1X PBS) by incubating for 30 min. at room temperature. The fixed silk glands were rinsed thrice with 1X PBS and mounted on a microscopic slide in Vectashield mounting medium with DAPI (Vector Labs), maintained in dark and were observed under a UV filter (excitation 340–380 nm) in a Fluorescent Microscope (Nikon Eclipse 80i) and images of overlapping fields were captured for cell enumeration.

### RNA preparation and mRNA isolation

The posterior and middle silk gland frozen tissues were homogenized in TRIzol Reagent (Invitrogen) for isolating total RNA. The resultant total RNA was purified by column adsorption using RNeasy Mini Kit (Qiagen). Multiple aliquots of 10 μg each of PSG and MSG total RNA were subjected to mRNA isolation using Poly(A)Purist MAG Kit (Ambion) by affinity chromatography using biotinylated oligo(dT) coupled to Streptavidin magnetic beads.

### Identification of partial sequences of A. assama H-fibroin

Next generation sequencing (Eurofins MWG Operon, Germany) of cDNA libraries prepared from MSG and PSG was performed. Briefly, poly(A) RNA was primed with random hexamers and was reverse transcribed for first strand synthesis. Then two unique adapters, A and B were ligated on 5′ and 3′ ends of the cDNA respectively and amplified with primers containing first four phosphorothioate-modified bases and 5′ biotinylated B-primer using a proofreading DNA polymerase. Reassociated ds-cDNA was separated from ss-cDNA by hydroxylapatite chromatography, and ss-cDNA was amplified for normalization. Library preparation and GS FLX Titanium sequencing of cDNA in the size range of 500–700 was done to obtain raw reads which were assembled to contigs. The contigs so obtained were merged with EST library sequences of *A. assama* PSG and MSG tissues from WildSilkBase and standalone BLASTn was performed using full length *A. pernyi* H-fibroin gene sequence as a query under general algorithmic parameters^15^. The specificity of the positive hits was checked through pairwise alignment with query sequences on CLUSTAL Omega program and by conceptual translation of the positive sequences^47^.

### Amplification and cloning of full length AaFhc

Pupa was homogenized in liquid Nitrogen using mortar and pestle, for extraction of genomic DNA by standard Phenol-Chloroform-Isoamyl alcohol method. Long PCR amplification was performed on gDNA template (50 ng for 50 μL reaction) with a hot start high-fidelity proofreading DNA polymerase, Phusion (Finnzymes) with GC buffer and 3% DMSO. Cycling program: initial denaturation of 98 °C, 30 s; 30 cycles of 98 °C, 10 s; 60 °C, 45 s; 72 °C, 5 min. and a final extension of 72 °C, 10 min. was used. Primers (AaF forward and reverse; Supplementary information 3) were designed to anneal on the 5′ and 3′ non-repetitious termini of the full length gene. Full length fibroin gene was cloned in pWKS30 using In-Fusion HD Cloning Kit (Clontech) and chemically transformed into ultra-competent *E. coli* (SURE strain; Stratagene) cells. The cells were spread-plated on LB-Amp plates (Amp and X-gal each, 100 μg/mL), incubated at room temperature for about 24 hours and positive colonies were screened by blue-white selection and confirmed by plasmid isolation and double-digestion release followed by sequencing the ends.

### Sequencing full length gene

For determination of full length sequence, restriction mapping through the combination of restriction digestion and partial digestion based sub-cloning and sequencing techniques were administered. Ligation reactions in sub-cloning were performed using Rapid Ligation Kit (Thermo Scientific). Sub-clones whose insert size was 1 kb or less were directly sequenced with M13 forward and reverse primers, while the clones with insert size larger than 1 kb were further restriction digested, followed by cloning and sequencing. Order of the sub-clones in full length gene was determined by analyzing junction sequences in partial digest clones and from the overlapping sequences of sub-clones through pairwise alignment by CLUSTAL Omega analysis and by conceptual translation.

### Isolating promoter sequence by inverse PCR

An aliquot of 200 ng of genomic DNA was restriction digested with BamHI and the digested DNA was diluted to 100 μL for overnight ligation at 4 °C. The resultant self-circularized DNA of 5 μL was used as template for inverse PCR (Primers - AaF_iPCR forward and reverse; Supplementary information 3) performed using EmeraldAmp GT PCR Master Mix (Takara) with cycling program: initial denaturation of 98 °C, 30 s; 30 cycles of 98 °C, 10 s; 60 °C, 30 s; 72 °C, 1 min. and a final extension of 72 °C, 10 min. was used.

### Isolating 3′ UTR by RACE

Sequence of cDNA between the translation stop and the poly(A) tail was determined by 3′ RACE using GeneRacer Kit (Invitrogen) following the manufacturer’s protocol. Amplification of the 3′ ends of cDNA was performed with EmeraldAmp GT PCR Master Mix (Takara) with cycling program: initial denaturation of 98 °C, 30 s; 30 cycles of 98 °C, 10 s; 60 °C, 30 s; 72 °C, 1 min. and a final extension of 72 °C, 10 min. Primers for 3′RACE were designed 251 bases upstream (*AaFhc*-3′RACE forward; Supplementary information 3) to the translation stop codon. Resultant products were gel-extracted, cloned and sequences were analysed.

### Analysis of fibroin gene sequence

Full length fibroin gene sequence and its conceptually translated sequence were analysed to elucidate its coding, non-coding, intronic, and coding regions; within CDS, crystalline and amorphous regions. Codon preference for major amino acids was calculated on Sequence Manipulation Suite^48^. The sequence conservation among fibroin gene sequences of different moths was analysed both by manual pairwise alignment and by CLUSTAL Omega program, and viewed using GeneDoc program to highlight conserved regions in the alignment. Phylogenetic analysis was performed by constructing cladogram on MEGA 6.0 analysis package using the neighbor-joining (NJ) method with the JTT matrix and pairwise gap deletion, with 1000 bootstrap replicates^49^. Signal peptide cleavage site analysis was performed by SignalP 4.1 program^50^. The crystalline region was analysed for repeats using conceptually translated CDS and conservation within repeats analysed by CLUSTAL Omega alignment of manually segregated repeat motifs and represented as sequence logos generated on WebLogo3 program^51^. Amino acid composition and Hydropathy index (GRAVY) was calculated using ExPASy ProtParam program. Kyte-Doolittle Hydropathy plot was generated using ExPASy ProtScale program and Hydropathy Analysis program^52^. Secondary structure prediction analysis was performed using SOPMA program^35^.

### Northern blotting

PSG and MSG specific poly(A) RNA aliquots of 500 ng each were denatured in three volumes of Formaldehyde loading buffer and were loaded on 1.0% agarose gel prepared with 1X MOPS buffer and 0.66 M Formaldehyde concentration. Resolved gel was blotted onto a nylon membrane (Amersham N+ Hybond) by wet capillary transfer in the medium of 1X MOPS buffer. Blotted membrane was UV-crosslinked and prehybridised by swirling in ULTRAhyb buffer (Ambion) at 42 °C for one hour and then hybridized overnight under high stringent conditions with α-^32^P (dATP) radiolabeled DNA probes synthesized by random priming on partial sequence clone of 1.6 kb towards 5′ side (clone h1) using Strip-EZ DNA Kit (Ambion). Two preliminary washes, 5 min. each were given with 2X SSC (Saline-Sodium citrate; 300 mM NaCl and 30 mM Na_3_.Citrate, pH 7.0), 0.1% SDS at room temperature followed by a couple of 30-minute high stringent washes with 0.1X SSC, 0.1% SDS at 42 °C. Wrapped wet membrane was exposed overnight against a phosphorscreen, and the image was captured on Starion FLA 9000 scanner (Fujifilm).

### Fibroin protein characterisation

Freshly spun cocoon shells of *A. assama* and *B. mori* were finely teased and boiled in 0.05% Na_2_CO_3_ aq. solution at 85 °C for 15 min, washed with several changes of hot MQ-water. Degummed cocoon silk was dried at 40 °C in a hot-air oven for weighing but directly forwarded after washing for solubilising. 50 mg (wet weight) of degummed cocoon silk was dissolved overnight by gentle rocking in 10 mL of 9M Lithium thiocyanate (Sigma) solution with 2% (v/v) β-Mercaptoethanol at room temperature. Then the silk solution was centrifuged at room temperature to remove any insoluble debris and the clear supernatant was solvent exchanged and concentrated to 100 μL at room temperature, using Amicon Ultra-15 (10 kDa cut-off) Centrifugal Filter (Millipore) with several volumes of 8M Urea (in 20 mM Tris-HCl; pH 8.0) with 2% (w/v) SDS and 5% (v/v) β-Mercaptoethanol. Solvent-exchanged fibroin concentrate samples were denatured for SDS-PAGE loading, in one volume of gel loading buffer: 10% SDS (Sigma), 50% Glycerol, 5% β-Mercaptoethanol, 0.5% Bromophenol Blue prepared in 250 mM Tris-HCl (pH 6.8), at 70 °C for 15 min and were loaded against a pre-stained heavy molecular weight protein ladder (Invitrogen Himark) on 5% stacking gel casted upon 5–15% linear gradient resolving gel, with 0.1% SDS both in gel and tank buffer, 1X TGS (Tris-Glycine-SDS) buffer^53^. The run was performed at room temperature with constant current, 25 mA on Hoefer SE600 electrophoresis unit. Protein bands were visualized by staining with Coomassie Brilliant Blue R-350 staining solution prepared from PlusOne Coomassie Tablets, PhastGel Blue R-350 (GE Healthcare Life Sciences).

## Additional Information

**How to cite this article**: Gupta K, A. *et al.* Molecular architecture of silk fibroin of Indian golden silkmoth, *Antheraea assama.* *Sci. Rep.* **5**, 12706; doi: 10.1038/srep12706 (2015).

## Supplementary Material

Supplementary Information

## Figures and Tables

**Table 1:** Biased usage of codons of major composite amino acid residues of *AaFhc* compared with other full length H-fibroins.

AA Residue	Codon	*A. assama*	*A. pernyi*	*A. yamamai*	*B. mori*
Ala	GCA	**67.3**	**58.2**	**57.5**	18.1
	GCC	3.4	6.7	9.0	10.4
	GCG	14.0	20.0	18.0	0.2
	GCU	15.3	14.9	15.4	**71.3**
Gly	GGA	30.3	26.6	28.1	39.4
	GGC	27.5	27.5	28.8	4.1
	GGG	**0.6**	**4.3**	**3.2**	**1.0**
	GGU	41.6	41.5	39.8	55.5
Ser	AGC	1.0	1.7	3.2	13.4
	AGU	9.0	11.4	10.2	1.9
	UCA	**69.7**	**57.5**	**61.0**	**68.0**
	UCC	7	5.1	1.6	0.6
	UCG	6.6	9.8	7.6	1.7
	UCU	6.6	14.5	16.5	14.3

The major amino acids of H-fibroin are represented in 3-letter codes and all their possible codons represented in the second column. The values against each isocodon represent the number of times it repeats in the coding sequence of *H-fibroin* corresponding to the organisms.

**Table 2:** Major composite amino acids of AaFhc and other full length H-fibroins.

Amino acid	*A. assama*		*A. pernyi*		*A. yamamai*		*B. mori*	
Ala	1193	42.5%	1137	43.1%	1122	42.3%	1592	30.2%
Gly	811	28.9%	720	27.3%	729	27.5%	2415	45.9%
Ser	287	10.2%	297	11.3%	292	11.0%	635	12.1%
Ile, Leu, Val	35	1.2%	42	1.6%	41	1.5%	117	2.2%
Arg, His, Lys	110	3.9%	99	3.8%	102	3.8%	31	0.6%
Asp, Glu	154	5.5%	135	5.1%	150	5.7%	55	1.0%
Tyr	138	4.9%	139	5.3%	136	5.1%	277	5.3%

Amino acid residues are represented in 3-letter codes and the values represent the number of residues and their percentages deduced from their conceptually translated full length H-fibroin sequences of the respective moth.
