# Supplementary material for: Molecular architecture of silk fibroin of Indian golden silkmoth, Antheraea assama
Source: Sci Rep. 2015 Aug 3;5:12706. doi: 10.1038/srep12706 (PMC4522600; doi:10.1038/srep12706)
Supplement: Supplementary Information [file srep12706-s1.doc]

***Supplementary information***

**Manuscript title: Molecular architecture of silk fibroin of Indian golden silkmoth, *Antheraea assama***

Author list: Adarsh Gupta K, Kazuei Mita, Kallare P Arunkumar and Javaregowda Nagaraju

**Supplementary information-1. AaFhc amino acid sequence with highlighted repeats.**

*MRVIAFVILCCVLQYATAKNIRHHDEYVDSNGQLFERFTTRKHFERNAETRPNLSGNERLVETIVLEEDPYGHENIYEEDVVIKRVPGASSSAAAASSASAGGRGGFYESHDSFVVDSSYGSSSSSS*AAAAAGSGAGGRGGGYGLGDGGYGSGSSAAAAAAAAAAAAGSGAGGAGDGGYGSGSSGAAAAAAAAAAAARRAGHDRAAGSAAAAAAAAAAAAAGSGAGGHGGGYGWGDDGSGSGSAAAAAAAAAAAAGSGAGGSGGGYGWGDEGYGSGSAAAAAAAAAAAAGSGAGGAGDGGYGSGSSGAAAAAAAAAAAAAARRAGHDRAAGSAAAAAAAAAAAAAGSGSGGYGGGYGWGDGGYGSDSAAAAAAAAAAAAAGSGAGGAGGGYGWGDEGYGSGSAAAAAAAAAAAAAGSGAGGSGGGYGWGDEGYGSGSAAAAAAAAAAAAGSGAGGAGDGGYGSGSSGAAAAAAAAAAAAAARRAGHDRAAGSAAAAAAAAAAAAAGSGAGGYGGGYGWGDGGYGSDSAAAAAAAAAAAAAGSGAGGSGGGYGWGDEGYGSGSAAAAAAAAAAAAGSGAGGSGGGYGWGDEGYGSGSAAAAAAAAAAAAGSGAGGAGDGGYGSGSSGAAAAAAAAAAAAAARRAGHDRAAGSAAAAAAAAAAAAAGSGAGGHGGGYGWGDGGYGSDSAAAAAAAAAAAAGSGAGGSGGGYGWGDDGYGSGSAAAAAAAAAAAAAGSGAGGAGDGGYGSGSSGAAAAAAAAAAAARRAGHDRAAGSAAAAAAAAAAAAGGSGAGGHGGGYGWGDGGYGSDSAAAAAAAAAAAAAGGSGAGGHGGGYGWGDGGYGSDSAAAAAAAAAAAAAGGSGGRGSGDGYGWGDGGYGSGSAAAAAAAAAAAAGSGAGGAGEGGYGSGSSGAAAAAAAAAAAAARRAGHDRAAGSAAAAAAAAAAAAAGSGAGGYGGGYGWGDGGYGSDSAAAAAAAAAAAAGSGAGGYGGGYGWGDGGYGSDSAAAAAAAAAAAAAGSGAGGSGDGYGWGDDSYGSGSAAAAAAAAAAAAAGSGAGGAGGGGYGSGSSGAAAAAAAAAAAARRAGHDRAAGSAAAAAAAAAAAAAGSGAGGHGGGYGWGDGGYGSDSAAAAAAAAAAAAAGSGGRGSGGGYGWGDGGYGSDSAAAAAAAAAAAAGSGAGGYGGGYGWGEDGSGSAAAAAAAAAAAAAGGSGAGGAGDGGYGSGSSGAAAAAAAAAAAAARRAGHDRAAGSAAAAAAAAAAAAGGSGAGGYDGGYGWGDGGYGSDSAAAAAAAAAAAAAGSGAGGYGGGYGWGDGGSSSGSAAAAAAAAAAAAGSGAGGAGDGGYGSGSSGAAAAAAAAAAAAAARRAGHDRAAGSAAAAAAAAAAAAAGSGAGGHGGGYGWGDGGYGSDSAAAAAAAAAAAAAGSGGRGSGGGYGWGDGGYGSDSAAAAAAAAAAAAAGSGAGGYGDGYGWGDGGSGSGSAAAAAAAAAAAAGSGAGGAGGGYGWGDEGYGSGSAAAAAAAAAAAAAAGSGAGGAGDGGYGSGSSGAAAAAAAAAAAAAAARRAGHDRAAGSAAAAAAAAAAAAGGSGAGGYGGGYGWGDGGYGSDSAAAAAAAAAAAAGSGAGGYGGGYGWGDGGYGSDSAAAAAAAAAAAAAGSGGRGSGGGYGWGDGGYGSGSAAAAAAAAGSGAGGSGGGYGWGDDGYGSGSAAAAAAAAAAAAAGSGAGGAGDGGYGSGSSGAAAAAAAAAAAAAARRAGHDRAAGSAAAAAAAAAAAAAGSGAGGYGGGYGWGDGGYGSDSAAAAAAAAAAAAAGSGAGGSGGGYGWGDEGYGSGSAAAAAAAAAAAAAAGSGAGGSGGGYGWGDEGYGSGSAAAAAAAAAAAAGSGAGGAGDGGYGSGSSGAAAAAAAAAAAAARRAGHDRAAGSAAAAAAAAAAAAAGSGAGGYGGGYGWGDGGYGSDSAAAAAAAAAAAAAGSGAGGSGGGYGWGDEGYGSGSAAAAAAAAAAAAAGSGAGGAGDGGYGSGSSGAAAAAAAAAAAAARRAGHDRAAGSAAAAAAAAAAAAAGSGGRGSGGGYGWGDGGYGSDSAAAAAAAAAAAAGSGAGGSGGGYGWGDDGYGSGSAAAAAAAAAAAAAGSGAGGAGDGGYGSGSSGAAAAAAAAAAAAARRAGHDRAAGSAAAAAAAAAAAAGGSGAGGYGGGYGWGDGGYGSDSAAAAAAAAAAAAGSGGRGSGDGYGWGDGGYGSDSAAAAAAAAAAAAAGSGAGGYGGGYGWGDGGSGSGSAAAAAAAAAAAGSGAGGAGGGYGWGDEGYGSGSAAAAAAAAAAAAAAGSGAGGAGDGGYGSGSSGAAAAAAAAAAAAAAARRAGHDRAAGSAAAAAAAAAAAAGGSGAGGYGGGYGWGDGGYGSDSAAAAAAAAAAAAGSGAGGYGGGYGWGDGGYGSDSAAAAAAAAAAAAGSGAGGVGGGYGRGDGGYGSGSSAAAAAAAAAAAGSGAGGSDGGYGWGDDGYGSGSAAAAAAAAAAAAAGSGAGGVGGGYGRGDGGYGSGSSAAAAAAAAAAAAARRAGHDRAAGSAAAAAAAAAAAAGSGAGGSGYGYGWDYEGYGSDSAAAAAAAAAAAAGSGGRGSGDGYGWGDGGYGSGSAAAAAAAAAAAGSGAGGSGDGYGWGDDGYGSGSAAAAAAAAAAAAGSGAGGAGGGYGRGDGGYGSGSSAAAAAAAAAAAARRAGYDRAHGAGSAAAAAAAAAAGPGATRPVGVYGSDDGFVLDGGYDSEGSAAAAAAAAAAAA*SSGARSAGGHPLLSICCKPCFHGHSYEASRISVH*

R-1 RRAGHDRAAGS

G_b_-10 GSGAGGSGGGYGWGDDGYGSGS

G_b_-11 GGSGAGGHGGGYGWGDGGYGSDS

G_b_-12 GGSGAGGHGGGYGWGDGGYGSDS

G_b_-13 GGSGGRGSGDGYGWGDGGYGSGS

G_b_-14 GSGAGGYGGGYGWGDGGYGSDS

G_b_-15 GSGAGGYGGGYGWGDGGYGSDS

G_b_-16 GSGAGGSGDGYGWGDDSYGSGS

G_b_-17 GSGAGGHGGGYGWGDGGYGSDS

G_b_-18 GSGGRGSGGGYGWGDGGYGSDS

G_b_-19 GSGAGGYGGGYGWGED--GSGS

G_b_-20 GGSGAGGYDGGYGWGDGGYGSDS

G_b_-21 GSGAGGYGGGYGWGDGGSSSGS

G_b_-22 GSGAGGHGGGYGWGDGGYGSDS

G_b_-23 GSGGRGSGGGYGWGDGGYGSDS

G_b_-24 GSGAGGYGDGYGWGDGGSGSGS

G_b_-25 GSGAGGAGGGYGWGDEGYGSGS

G_b_-26 GGSGAGGYGGGYGWGDGGYGSDS

G_b_-27 GSGAGGYGGGYGWGDGGYGSDS

G_b_-28 GSGGRGSGGGYGWGDGGYGSGS

G_b_-29 GSGAGGSGGGYGWGDDGYGSGS

G_b_-30 GSGAGGYGGGYGWGDGGYGSDS

G_b_-31 GSGAGGSGGGYGWGDEGYGSGS

G_b_-32 GSGAGGSGGGYGWGDEGYGSGS

G_b_-33 GSGAGGYGGGYGWGDGGYGSDS

G_b_-34 GSGAGGSGGGYGWGDEGYGSGS

G_b_-35 GSGGRGSGGGYGWGDGGYGSDS

G_b_-36 GSGAGGSGGGYGWGDDGYGSGS

G_b_-37 GGSGAGGYGGGYGWGDGGYGSDS

G_b_-38 GSGGRGSGDGYGWGDGGYGSDS

G_b_-39 GSGAGGYGGGYGWGDGGSGSGS

G_b_-40 GSGAGGAGGGYGWGDEGYGSGS

G_b_-41 GGSGAGGYGGGYGWGDGGYGSDS

G_b_-42 GSGAGGYGGGYGWGDGGYGSDS

G_b_-43 GSGAGGSDGGYGWGDDGYGSGS

G_b_-44 GSGAGGSGYGYGWDYEGYGSDS

G_b_-45 GSGGRGSGDGYGWGDGGYGSGS

G_b_-46 GSGAGGSGDGYGWGDDGYGSGS

G_c_-1 GSGAGGRGGGYGLGDGGYGSGSS

G_c_-2 GSGAGGVGGGYGRGDGGYGSGSS

G_c_-3 GSGAGGVGGGYGRGDGGYGSGSS

G_c_-4 GSGAGGAGGGYGRGDGGYGSGSS

R-2 RRAGHDRAAGS

R-3 RRAGHDRAAGS

R-4 RRAGHDRAAGS

R-5 RRAGHDRAAGS

R-6 RRAGHDRAAGS

R-7 RRAGHDRAAGS

R-8 RRAGHDRAAGS

R-9 RRAGHDRAAGS

R-10 RRAGHDRAAGS

R-11 RRAGHDRAAGS

R-12 RRAGHDRAAGS

R-13 RRAGHDRAAGS

R-14 RRAGHDRAAGS

R-15 RRAGHDRAAGS

R-16 RRAGHERASGS

G_a_-1 GSGAGGAGDGGYGSGSSG

G_a_-2 GSGAGGAGDGGYGSGSSG

G_a_-3 GSGAGGAGDGGYGSGSSG

G_a_-4 GSGAGGAGDGGYGSGSSG

G_a_-5 GSGAGGAGDGGYGSGSSG

G_a_-6 GSGAGGAGEGGYGSGSSG

G_a_-7 GSGAGGAGGGGYGSGSSG

G_a_-8 GSGAGGAGDGGYGSGSSG

G_a_-9 GSGAGGAGDGGYGSGSSG

G_a_-10 GSGAGGAGDGGYGSGSSG

G_a_-11 GSGAGGAGDGGYGSGSSG

G_a_-12 GSGAGGAGDGGYGSGSSG

G_a_-13 GSGAGGAGDGGYGSGSSG

G_a_-14 GSGAGGAGDGGYGSGSSG

G_b_-1 GSGAGGHGGGYGWGDDGSGSGS

G_b_-2 GSGAGGSGGGYGWGDEGYGSGS

G_b_-3 GSGSGGYGGGYGWGDGGYGSDS

G_b_-4 GSGAGGAGGGYGWGDEGYGSGS

G_b_-5 GSGAGGSGGGYGWGDEGYGSGS

G_b_-6 GSGAGGYGGGYGWGDGGYGSDS

G_b_-7 GSGAGGSGGGYGWGDEGYGSGS

G_b_-8 GSGAGGSGGGYGWGDEGYGSGS

G_b_-9 GSGAGGHGGGYGWGDGGYGSDS

**Supplementary information-2. Conservation within non-polyalanine motifs of AaFhc. The repeats are numbered according to their position in AaFhc molecule. Conservation among the repeats is shown with black background and gray background for the less conserved residues.**

**Supplementary information-3. Primer sequences**

a) Full length Long PCR Primers:

AaFhc forward: 5’- TGGTCTCATTATCAGTTCGGTTCCAGC -3’

AaFhc reverse: 5’- TTAGTGGACGGAAATTCTGGAAGCTTC -3’

b) In-Fusion Cloning Primers:

AaFhc_pWKS30 forward: 5’- CTGCAGGAATTCGATATCGTCTCATTATCAGTTCGGTTCCAG -3’

AaFhc_pWKS30 reverse: 5’- ATCGATAAGCTTGATATCTTAGTGGACGGAAATTCTGGAAG 3’

c) Primers Used for iPCR:

AaFhc_iPCR forward: 5’- CGACATTCATTTAAATGATTCCATGG -3’

AaFhc_iPCR reverse: 5’- CCAGATCACGAAGGCTATTACTCTCA -3’

d) Primers Used in 3’ RACE

GeneRacer Oligo dT Primer: 5’- GCTGTCAACGATACGCTACGTAACGGCATGACAGTG(T)_18_ -3’

GeneRacer 3’ Primer: 5’- GCTGTCAACGATACGCTACGTAACG -3’

GeneRacer 3’ Nested Primer: 5’- CGCTACGTAACGGCATGACAGTG -3’

AaFhc-3’RACE forward: 5’- TGCATATGGAGCTGGAAGTG -3’
